# When Will the Lockdown End? Confinement Duration Forecasts and Self-Reported Life Satisfaction in Spain: A Longitudinal Study

**DOI:** 10.3389/fpsyg.2021.635145

**Published:** 2021-03-24

**Authors:** Gerardo Sabater-Grande, Aurora García-Gallego, Nikolaos Georgantzís, Noemí Herranz-Zarzoso

**Affiliations:** ^1^LEE & Economics Department, Universitat Jaume I, Castellón de la Plana, Spain; ^2^CEREN EA 7477, Burgundy School of Business, Université Bourgogne Franche-Comté, Dijon, France

**Keywords:** COVID-19, lockdown, personality traits, gender, daily life satisfaction

## Abstract

This paper reports results from a longitudinal study on the impact of the lockdown on daily self-reported life satisfaction levels during the first wave of the COVID-19 pandemic in Spain. A stable panel (*N* = 1,131) of adult subjects were surveyed during 84 consecutive days (March 29–June 20, 2020). They were asked to report daily life satisfaction and health state levels. Interestingly, daily life satisfaction increased during the lockdown. At the beginning of the experiment, subjects were asked to guess the end-week of the lockdown, against a possible monetary reward for accurate forecasts. Subjects predicting a longer lockdown period reported a higher average level of daily life satisfaction. Females reported on average lower levels of daily life satisfaction, but exhibited a stronger tendency to report higher levels of life satisfaction, the longer their lockdown forecast. Individual heterogeneity in life satisfaction levels can be partly attributed to personality traits, with neuroticism having a negative effect, while extraversion and agreeableness having a positive effect on daily life satisfaction.

## Introduction

On January 30, 2020, the World Health Organization (WHO) announced that the severe acute respiratory syndrome coronavirus 2 (SARS-CoV-2; COVID-19, hereafter) had developed into a global health emergency. On March 11, 2020, the WHO declared COVID-19 a global pandemic. Currently, COVID-19 has infected over 110 million people and resulted in over 2.1 million deaths worldwide (WHO, 2020). By the end of February, 2021, ~3,153,971 COVID-19 cases have been officially reported in Spain, over a population of 46,348,000. At least 67,636 have died due to the virus since January 3, 2020, when the first case was reported.

Moreover, given that, at the moment this article is being written, there is no effective drug for the treatment of COVID-19, whereas the vaccines developed will not be broadly available in most countries until the Spring of 2021, social distancing and gentle rule enforcement has been the most effective measures with which governments try to control the outbreak. In Spain, the state of emergency was declared on March 14, 2020, imposing a “lockdown,” during which people had to stay at home, allowed to go out only for work, buying food and satisfying essential needs like physical exercise at a maximal distance of 1 km from their permanent address. Further, starting March 30, all workers of non-essential activities were obliged to stay home until April 9. These confinement measures were progressively softened until June 21, when mobility restrictions were waived^1^.

Although limiting physical contact among people is paramount in order to protect them from the pandemic, the lockdown has been often blamed for a deterioration in life quality^2^. According to the meta-analysis by Salari et al. (2020), the recent lockdown due to COVID-19 may negatively affect people's mental health. In China, depression and anxiety effects were reported in college students (Cao et al., 2020; Zhou et al., 2020), university students (Wang Y., et al., 2020) and working adults (Zhang et al., 2020) at early stages of the COVID-19 outbreak. Wang Y., et al. (2020), Huang and Zhao (2020), Wang C. et al. (2020), Ahmed et al. (2020), Zhou et al. (2020), and Liu et al. (2020) reported increasing levels of depression because of the COVID-19 pandemic, ranging between slightly less than one fifth and half of the samples surveyed. These papers, together with Cao et al. (2020) and Qian et al. (2020) report percentages of people suffering from anxiety which range between 6 and 44%.

Regarding the relationship between sociodemographic variables and the psychological costs of COVID-19 in China, the results are mixed. Some studies have suggested than women were affected psychologically by COVID-19 more than men (Qiu et al., 2020; Wang C. et al., 2020; Wang Y., et al., 2020) but others found no significant differences between females and males (Cao et al., 2020; Huang and Zhao, 2020; Qian et al., 2020; Yang and Ma, 2020). Related to age, results obtained by Huang and Zhao (2020), Liu et al. (2020), Qiu et al. (2020), and Wang Y, et al. (2020) suggest that young subjects exhibit higher psychological costs than mature people, although results vary across studies^3^. In China, education levels have been found to positively (Qiu et al., 2020) or negatively (Wang Y., et al., 2020) correlate with the psychological costs, while also no effect has been reported (Qian et al., 2020). Finally, chronic disease has been found to enhance negative psychological effects during lockdown (Wang C. et al., 2020).

Concerning life satisfaction, Yang and Ma (2020) found that the pandemic in China led to a 74% decline in overall emotional well-being. Individuals who were residing near the epicenter of the outbreak, of an older age, or married, experienced a steeper decline in emotional well-being. Furthermore, Zhang et al. (2020) obtained that physically active subjects reported lower levels of life satisfaction due to the restrictions imposed.

Other Asian countries were also affected and studied. In Iran, Jahanshahi et al. (2020) found that adults experienced more distress than those in China. Moghanibashi-Mansourieh (2020) obtained that the level of anxiety was higher among Iranian women, COVID-19 informed people and younger adults. Results about gender and education are replicated for the Iraq population (Kamal and Othman, 2020) and for Nepal (Sigdel et al., 2020). Age results are mirrored for Japanese people (Ueda et al., 2020) and Indians (Kazmi et al., 2020). Other variables as perceived health conditions and perceived COVID-19 test availability are predictors of mental health problems for Malaysia citizens in Dai et al. (2020).

United Kingdom, Italy and Spain were three of the most affected European countries. In United Kingdom, Shevlin et al. (2020) analyzed the association between self-reported anxiety caused by COVID-19 and somatization, finding that anxiety contributes to experiencing somatic symptoms. In Italy, Mazza et al. (2020) found that higher levels of depression, anxiety, and stress were associated with female gender and the personality domains of negative affect and detachment.

In Spain, Odriozola-González et al. (2020a) conducted a cross-sectional study where anxiety, depression and stress were observed in 32, 44, and 37% of respondents, respectively. The prevalence of these symptoms was associated with female gender, younger age and self-reported COVID-19 symptoms. In a companion paper, Odriozola-González et al. (2020b) analyze the psychological impact of COVID-19 in the university community, finding that students have been especially impacted by the first weeks of the COVID-19 confinement. Similar results were reported by et al. (2020) who explored the psychological impact of the COVID-19 pandemic in the general adult population during the first stages of the outbreak in Spain. Moreover, in line with Shevlin et al. (2020) subjects' perceived health state was negatively associated with psychological impact, stress, anxiety, and other symptoms related to depression.

Finally, Blasco-Belled et al. (2020) investigated reactions to the COVID-19 outbreak and the impact on subjective well-being in a sample of 541 Spanish adults. They found that life satisfaction was predicted positively by hope about overcoming the pandemic and negatively by social phobia given that human-to-human contact can be perceived as a source of potential danger to be avoided.

Extending the aforementioned research on the psychological impact of COVID-19 in Spain, the present study examined the temporal evolution of life satisfaction during the lockdown from the first phase of the pandemic to the slow return to a new “normality.” With the exceptions of Stuchlikova et al. (2020) and Planchuelo-Gómez et al. (2020), where data concerned four and two time-frames, respectively, to our knowledge, this is the only study generating empirical evidence on daily life satisfaction over the whole period of the lockdown. Following the experience sampling method^4^, we ask participants to repeatedly self-report their feelings. De Vuyst et al. (2019) study whether using this method can in some way affect the momentary emotional self-reports of the individuals engaging in them, and thereby effectively affecting that what it tries to measure: their emotional experience over time. Their findings suggest that the repeated assessment of emotions in daily life does not influence their emotional experience throughout the response period. Moreover, this is the first paper analyzing the role of individual (incentivized) expectations about the lockdown length on the evolution of subjects' life satisfaction.

Additionally, since “many studies have highlighted significant differences in individuals' reactions to threat, according to specific personality traits” (Mazza et al., 2020) we have analyzed how subjects' personality can explain perceived life satisfaction and its evolution during lockdown.

## Methods

### Participants and Procedure

An online experiment (Googleforms-platform) was carried out, using the online recruitment process (ORSEE) usually adopted to recruit student subjects at the Laboratorio de Economía Experimental (LEE) of the Universitat Jaume I (UJI) in Castellón (Spain)^5^. At the beginning of the experiment, 1,131 adult volunteers were recruited. They were first incentivized to accurately predict the end-week of the lockdown from a set of available options (from April 11 to May 23 in 1-week intervals, including the option “beyond May 23”). Participants were informed that 20 prizes of €200 would be randomly drawn among those submitting correct predictions. In order to let the incentives for participants to remain active, once each of the six earliest dates available were exceeded, subjects were given a new chance to predict the lockdown's end-week^6^. Ten new prizes of €200, were added to reward randomly subjects among those submitting a correct prediction in the second trial. The following “second-chance” predictions were offered: In a second wave, after predictions of April 11 and April 18 had failed, 72 out of 131 subjects chose from a series of choices (from May 2 to May 23 in 1-week intervals, including the option “beyond May 23”); in a third wave, after expectations of April 25 and May, 2 had been unsuccessful, 364 out 498 selected one new option (from May 9 to June 20 in 1-week intervals, including the option “beyond June 20”); in a fourth wave, after predictions of May 9 and May 16 had failed, 234 out 338 participants responded among the following proposed lockdown end date: from May 23 to July, 4, including the option “beyond July 4.” Only one second-chance prediction was allowed for each subject. Regarding options offered in order to predict the end of the lockdown all possibilities should be covered including the more pessimistic expectations. In order to offer an acceptable number of prospects we opted for including an uncertain and distant date of the end of a lockdown. It might affect negatively subjects' well-being due to the lack of control over what might happen in the future. As opposed, although offering only closed estimations about the end of the confinement could restore a bit of control to the individual, it can suggest ideas to some respondents and frustrating to others because their desired answer is not an option.

Socio-demographic characteristics, general life satisfaction (using the life satisfaction scale, SWLS) and personality traits (by means of a NEO-FFI test) were also obtained. Moreover, from March 30 to June 20, subjects were asked on a daily basis about their current life satisfaction and health state. Finally, twice a week, subjects were asked: (1) to report whether they experienced any COVID-19 symptoms; (2) their willingness to test for COVID-19 with no payment accepting a 14-day confinement in case of a positive result; (3) their willingness to pay 150€ in order to test privately for COVID-19 with a non-mandatory confinement in case of a positive result. Prior to data collection, informed consent had been obtained from all subjects acknowledging acceptance of our data management protocols involving anonymity, confidentiality, and exclusive use for the present scientific research.

The average age of subjects in the sample was 27.36 years old (SD = 10.39) and 40% were men. Regarding education, 1% had completed primary education, 10% secondary education, 8% professional training, and 68% a university degree, whereas 10% had a master's degree and 2% a PhD. Related to their home conditions during lockdown, the average home surface of the sample was 132.04 m^2^ (SD = 137.91). Finally, 86% of subjects reported no chronic health problems at the beginning of the study.

### Measures

#### Life Satisfaction Measure

Life satisfaction was measured by the Satisfaction with life Scale (SWLS; Diener et al., 1985), probably the measure of life satisfaction which is the most cited in the scientific literature (Diener et al., 2013). This scale comprises five self-referencing statements on global life satisfaction. Participants completed the Spanish version of the SWLS (Atienza et al., 2003).

#### Personality Data

We used the short form of the NEO Personality Inventory-Revised (NEO-PI-R), the NEO-FFI (Costa and Robert McCrae, 1992), in order to assess personality dimensions according to the Five Factor approach to personality. The NEO-FFI contains 60 items in the form of statements, to which participants are asked to rate their agreement, using a five-point Likert scale ranging from 0 (strongly disagree) to 4 (strongly agree). The NEO-FFI scales yield scores for the following personality domains (traits): Neuroticism, as a tendency to experience negative emotions and psychological distress in response to stressors; Extraversion, as a degree of sociability, positive emotionality, and general activity; Openness to Experience, as levels of curiosity, independent judgment, and conservativeness; Agreeableness, as altruistic, sympathetic, and co-operative tendencies; Conscientiousness, as one's level of self-control in planning and organization.

#### Repeated Lockdown Questionnaire

##### Lockdown Daily Questions

During the lockdown, we asked subjects to respond to two 5-point Likert scale questions:

1) Daily Question 1 (DQ1): How satisfied are you with your life in general?

Possible responses were: 1 (very dissatisfied), 2 (dissatisfied), 3 (neither), 4 (satisfied), 5 (very satisfied).

2) Daily Question 2 (DQ2): In general, how is your health?

Responses allowed were: 1 (very bad), 2 (bad), 3 (average), 4 (good), 5 (very good).

##### Lockdown Semi-Weekly Questions

During the entire lockdown period, every Monday and Friday, subjects were asked to reply with “Yes” or “No,” to the question:

(SQ1): Have you developed any symptoms compatible with COVID-19 that could make you think you have been infected?

Also, a four-point Likert scale (1: definitely won't; 2: probably won't; 3: probably will; 4: definitely will) was used to respond to the questions:

(SQ2): Would you be willing to be tested for COVID-19 for free by the health authorities knowing that a positive result would imply a mandatory confinement during 14-days?

and

(SQ3): Would you be willing to pay €150 to get tested for COVID-19 privately, leaving the consequences of a possible positive to your choice?

## Data Analyses and Results

In this section, we conduct descriptive analysis of the main variables. Additionally, we present figures showing the evolution of the main variables, like for instance the prediction of the lockdown end. After that, we present regression analysis, focusing first on the effect of lockdown-end expectation on reported daily life satisfaction. We then focus on participants' bias regarding the duration of the confinement. Lastly, we study the participants' willingness to be tested either for free or privately, paying for the test.

### Descriptive Statistics

In Table 1, we present descriptive statistics (mean and standard deviation) of the variables included in our study for the total population, men, and women. Moreover, we assess median differences between men and women using a Mann-Whitney *U*-test. Women presented higher scores not only in Neuroticism and Agreeableness, as usual, but also in Openness and Conscientiousness. However, previous findings reporting that women scored higher in Extraversion were not confirmed for our sample^7^. In addition, results of the SWLS questionnaire revealed that, like in Joshanloo and Jovanović (2020), women reported higher levels of overall life satisfaction. In our sample, women reported a worse chronic health condition than men and also predicted a longer lockdown. Finally, during the lockdown, women reported lower levels of daily life satisfaction and health state. They also had a higher willingness to test for COVID-19.

**Table 1:** Means, standard deviations, and Mann-Withney *U*-test values for gender differences in the variables included in the study.

	**Total (*****N*** **=** **1183)**		**Men (*****M*** **=** ***471*****)**		**Women (*****F*** **=** **712)**		**Median differences**
	**M**	**SD**	**M**	**SD**	**M**	**SD**	
Lockdown prediction (in weeks)	4.59	1.93	4.27	1.91	4.79	1.92	−4.527***
Age	27.36	10.39	28.14	10.92	26.84	10.00	3.983***
Chronic health disease	1.86	0.34	1.90	0.30	1.84	0.37	2.900***
Home surface	132.04	137.91	137.64	172.81	128.32	108.83	0.559
Neuroticism	20.79	8.52	17.83	7.77	22.74	8.43	−9.713***
Extraversion	30.52	6.88	30.32	6.88	30.66	6.88	−0.521
Openness	28.95	6.40	28.31	6.24	29.37	6.47	−2.580***
Agreeableness	31.98	5.99	30.90	6.02	32.70	5.87	−5.177***
Conscientiousness	32.03	7.47	30.77	7.44	32.86	7.39	−4.701***
SWLS-1	3.61	0.91	3.57	0.89	3.64	0.93	−1.648*
SWLS-2	3.70	0.87	3.69	0.83	3.71	0.90	−1.134
SWLS-3	3.84	0.78	3.85	0.74	3.83	0.81	0.179
SWLS-4	3.77	0.91	3.66	0.96	3.83	0.87	−3.268***
SWLS-5	3.20	1.16	3.04	1.15	3.29	1.15	−3.669***
DQ1	3.94	0.66	3.99	0.61	3.89	0.69	2.071**
DQ2	4.24	0.61	4.34	0.59	4.17	0.62	4.598***
SQ1	1.14	0.32	1.14	0.32	1.14	0.31	0.440
SQ2	3.67	0.60	3.62	0.65	3.70	0.56	−2.012**
SQ3	1.89	0.89	1.81	0.87	1.94	0.89	−2.506**

*SWLS-1: In most ways my life is close to my ideal; SWLS-2: The conditions of my life are excellent; SWLS-3: I am satisfied with my life; SWLS-4: So far I have gotten the important things I want in life; SWLS-5: If I could live my life over, I would change almost nothing; DQ1: How satisfied are you with your life in general? DQ2: In general, how is your health? SQ1: Have you developed any symptoms compatible with COVID-19 that could make you think you have been infected? SQ2: Would you be willing to be tested for COVID-19 for free by the health authorities knowing that a positive would imply a mandatory 14-days confinement? SQ3: Would you be willing to pay €150 to get tested for COVID-19 privately, leaving the consequences of a possible positive to your choice? Median differences using a Mann-Whitney U-test*.

Levels of significance: ***p < 0.01, **p < 0.05, *p < 0.10.

**Figure 1:**
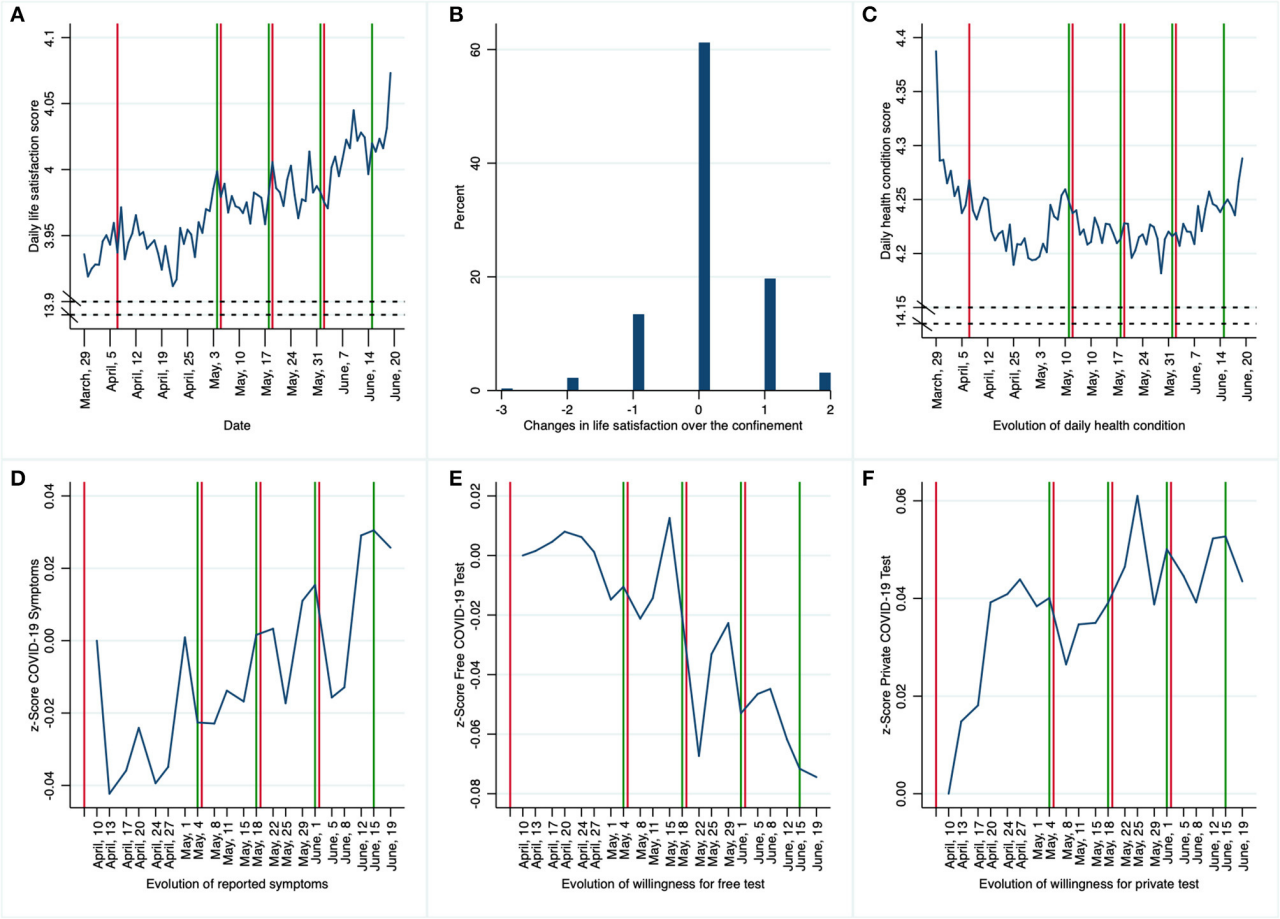
Evolution of responses to daily **(A,C)** and semiweekly **(D–F)** questions and overall change in life satisfaction **(B)** during lockdown from March 29 to June 20, along with dates in which the state of alarm was extended (in red) and initial dates in the de-escalation four-phase plan (in green).

In Figure 1, we present the evolution of subjects' responses to the five questions repeated during lockdown from March 29 to June 20, including overall change in life satisfaction from day 1 to day 84, along with dates in which the state of alarm was extended (in red) and initial dates in the de-escalation four-phase plan (in green)^8^.

We can observe in Figure 1A that, in general, the daily average life satisfaction is surprisingly high during the confinement (around 4, indicating that subjects are satisfied with her life). This score increases when the initial day of phase 0 (first green line) is announced, and especially at the beginning of the intermediate phase (phase 2). These results contrast to Planchuelo-Gómez et al. (2020), who reported increased levels of anxiety, depression and (especially) stress in May 2020 with respect to April of the same year.

Figure 1B shows the percentage of changes in life satisfaction scores over the confinement. It is observed that slightly over 60% of the subjects reported the same life satisfaction level at the beginning and at the end of the lockdown. More than 20% of the participants reported higher levels of life satisfaction at the end, while fewer reported lower levels toward the end, with only a small group among them exhibiting a maximal drop of three grades on the Likert scale.

Figure 1C displays the evolution of the average daily reported score on health condition during the confinement. In the course of the lockdown, average scores are well above 4, corresponding to a good health condition. After an accelerated drop at the initial stage, the subjects' health condition remains constant until the intermediate phase, in which the average score increases again.

In Figure 1D, z-scores obtained from the “Have you developed any symptoms compatible with COVID-19 …” question (“Yes/No” transformed into “1-2”) standardized using the first period mean and standard deviation. This makes it easier to follow the change over time with a clear representation of the effect size. We observe that the standardized response to this question is under 0.05 standard deviations below/above the first-period average. Hence, a rather stable temporary profile is obtained, presenting a slight increase since the announcement of the de-escalation plan.

Similar to Figures 1D,E shows z-scores obtained from the “Would you be willing to be tested for COVID-19 for free…” question (“definitely won't/probably won't/probably will/definitely will” transformed into “1-2-3-4”) standardized using the first period mean and standard deviation. A decreasing trend is observed, as long as the de-escalation advances and restrictions are relaxed (the average response regarding willingness to be COVID-19- tested for free at the end of the confinement is 0.08 standard deviations below the aforementioned average in the first period).

Similar to previous figures, in Figure 1F we present z-scores obtained from the “Would you be willing to pay €150 to get tested for COVID-19…” question (“definitely won't/probably won't/probably will/definitely will” transformed into “1-2-3-4”) standardized using the first period mean and standard deviation. A rather stable pattern is observed over time, only showing a slight increase since the announcement of the de-escalation plan (when the average willingness to be privately tested for COVID-19 in each period is up to 0.06 standard deviations above the aforementioned average in the first period).

**Figure 2:**
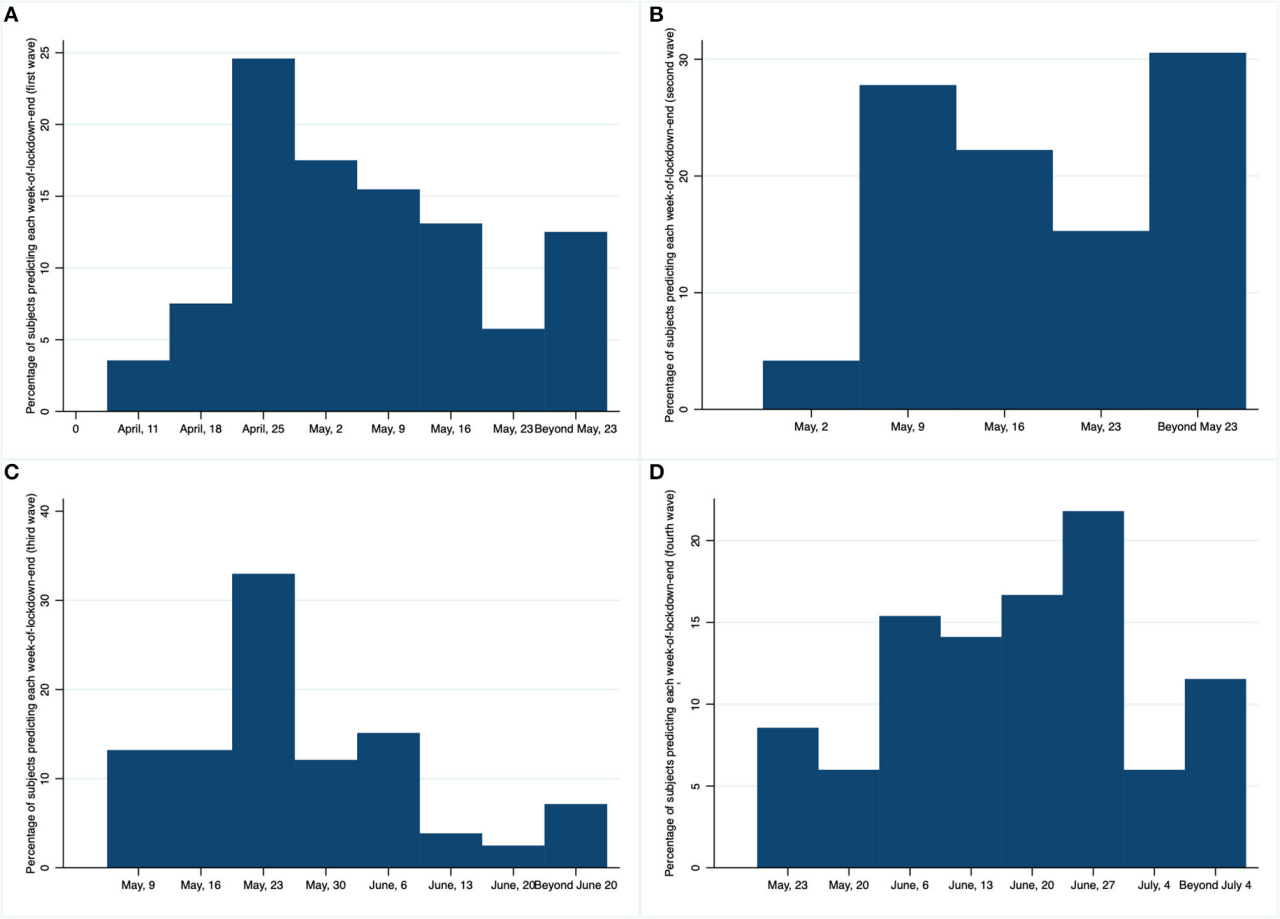
Percentage of subjects predicting each week-of-lockdown-end in the four (**A–D**) lockdown-end prediction elicitation waves.

Figure 2 presents percentages of subjects' weekly prediction of week-of-lockdown-end for each wave. We can observe in Figure 2A that, in the first wave, only 13% of our sample predicted correctly the duration of the state of alarm (including options from April 11 to May 23, and “beyond May 23”). In fact, the average underestimation (difference between the real end of the lockdown and the prediction) is equal to 42.27 days. This widespread mismatch between people's predictions and real events can be due to the lack of transparency in government's announcements and, thus, failure to manage people's expectations^9^. Contrary to Tetlock and Gardner (2016), we do not find that good forecasters score higher in openness to experience than unsuccessful predictors^10^.

After predictions of April 11 and April 18 had failed, unsuccessful participants (30% of them predicted the first date and 70% chose the second option) were given a new incentivized chance to predict the lockdown's end-week^11^. In Figure 2B, it is shown how the percentage of successful predictions in the second wave rises from <13% to slightly above 30%. Moreover, in average the underestimation for these subjects is significantly lower (27.76 days) in the second wave than in the first wave (64.17 days).

After expectations of April 25 and May 2 were proven wrong, another group of participants (59% of them selected the first date and 41% preferred the second choice) were allowed to have a second chance to predict the end of the confinement^12^. Figure 2C presents the percentage of subjects by proposed date. In average, participants included in this third wave underestimate the duration of the lockdown by 26.18 days which is as expected lower than a stronger previous bias (53.10 days).

Lastly, after predictions of May 9 and May 16 had failed, a fourth (and final) wave of predictions were submitted (55% of the new unsuccessful participants opted for the first date and 45% preferred the second choice). Although a slight overestimation appears for the first time in our study, we focus on the underestimation phenomenon. Figure 2D displays the percentage of subjects choosing each new expected lockdown-end date. On average, participants included in this last wave underestimated the duration of the lockdown only by 11.19 days, instead of 38.75 days corresponding to the first prediction.

### Regression Analysis

In order to account for the effect of lockdown-end expectations on daily life satisfaction, we run two Tobit models. Results corresponding to the first and the second predictions are reported in Table 2. In the model on the left-hand-side column, all lockdown predictions were elicited at the beginning of the study. In the right-hand-side column model, lockdown predictions were elicited at the beginning of the study (for subjects making only one prediction) and also in the course of the lockdown (for subjects submitting a second prediction).

**Table 2:** Tobit models.

	**DQ1**	**DQ1**
	**First**	**Second**
Lockdown prediction	0.0258^**^ (0.0128)	0.00203 (0.0057)
Gender	−0.295^***^ (0.0819)	−0.179^***^ (0.0577)
Education	0.0295 (0.0183)	0.0302^*^ (0.0183)
Predic*gender	0.0409^**^ (0.0164)	0.0161 (0.011)
Neuroticism	−0.0055^**^ (0.0023)	−0.0056^**^ (0.0023)
Extraversion	0.0100^***^ (0.0025)	0.0102^***^ (0.0025)
Openness	−0.0020 (0.0025)	−0.002 (0.0025)
Agreeableness	0.0061^**^	0.0060^**^
	(0.0029)	(0.0029)
Consciousness	0.0027 (0.0023)	0.0028 (0.0023)
SWLS-1	0.0961^***^ (0.0239)	0.0936^***^ (0.0240)
SWLS-2	0.0648^***^ (0.0233)	0.0642^***^ (0.0234)
SWLS-3	0.201^***^ (0.0300)	0.204^***^ (0.0301)
SWLS-4	0.0406^*^ (0.0208)	0.0403^*^ (0.0208)
SWLS-5	0.0732^***^ (0.0158)	0.0735^***^ (0.0158)
Health condition	0.154^***^ (0.0588)	0.154^***^ (0.0589)
Chronic disease	0.0032 (0.0642)	0.0005 (0.0643)
Home Surface	4.45e-07 (0.0001)	1.84e-06 (0.0001)
Age	0.0026 (0.0016)	0.0024 (0.0016)
Constant	1.253^***^ (0.224)	1.352^***^ (0.220)
Observations	1131	1131

*Dependent variable: daily life satisfaction*.

*SWLS-1: In most ways my life is close to my ideal; SWLS-2: The conditions of my life are excellent; SWLS-3: I am satisfied with my life; SWLS-4: So far I have gotten the important things I want in life; SWLS-5: If I could live my life over, I would change almost nothing*.

*Standard errors in parentheses*.

*** *p < 0.01*,

** *p < 0.05*,

* *p < 0.1*.

*Tobit model DQ1 First: Wald test (19, 1113) = 3569.45. Tobit model DQ1 Second: Wald test (19, 1113) = 3556.88*.

The variables used in the models are the following:

- Daily reported life satisfaction (DQ1): dependent variable. Ranges from 1 to 5.
- First lockdown prediction: incentivized expectation on the duration of the state of alarm at the beginning of the study. Ranges from “1 week” to “8 weeks” in 1-week intervals, including a “more than 8 weeks” option.
- Second lockdown prediction: incentivized expectation of the duration of the state of alarm at the three waves carried out during the lockdown. Ranges from “1 week” to “7 weeks” in 1-week intervals, including a “more than 8 weeks” option.
- Education: ranges from 0 (primary education) to 6 (PhD).
- Gender: takes value 1 if the subject is female, 0 otherwise.
- Age: reported by the subjects (in years) at the beginning of the study.
- Home surface: number of squared meters where a subject lives during the confinement.
- Satisfaction with Life scale (SWLS): five self-reported statements on global life satisfaction. Ranges from 1 (totally disagree) to 5 (totally agree) in each statement.
- Health condition: dummy variable taking the value 1 for subjects reporting any type of health problem at the beginning of the study, 0 otherwise.
- Predic^*^gender: interaction between gender and lockdown prediction. It captures the lockdown prediction by women.
- Chronic disease: dummy variable that takes the value 1 for subjects reporting a chronic health problem at the beginning of the study, and 0 otherwise.
- Five dimensions of personality (Neuroticism, Extraversion, Openness, Agreeableness, Consciousness), 12 items per domain: each item ranges from 0 (strongly disagree) to 4 (strongly agree).

We find that a longer lockdown expectation at the beginning of the study affects positively the average daily life satisfaction. This is probably due to disappointment and frustration experienced by subjects predicting a shorter confinement period. When confinement-end expectations are negatively surprised by extensions of the state of alarm, participants report a significant lower level of life satisfaction. This finding is supported by the fact that the effect of “lockdown prediction” on daily life satisfaction vanishes when revised expectations are considered, once their initial forecast has failed, as can be observed in the right column model. Of course, our study cannot disentangle this from other underlying factors leading to the negative correlation between daily life satisfaction and forecasted lockdown duration, but in any case, we can certainly exclude optimism or underestimation of the severity of the pandemic, which should have produced the contrary effect, namely, associating shorter lockdown-end forecasts with higher levels of daily life satisfaction.

Not surprisingly, average daily life satisfaction during the lockdown is related to a subject's global life satisfaction level, reported through the SWLS. Moreover, even though chronic afflictions do not seem to affect daily life satisfaction levels, subjects acknowledging non-chronic health problems report a lower daily life satisfaction.

Regarding personality traits, we find that, whereas neuroticism has a negative effect on average daily life satisfaction reported during the lockdown, domains like extraversion and agreeableness have a positive effect. Gender differences are observed, with females reporting lower daily life satisfaction levels than males. The interaction effect included in the regression using only the first predictions informs us that women predicting a longer lockdown at the beginning of the experiment are better off than men. This effect is not significant when first predictions are replaced by second predictions.

In order to explain the lockdown under-estimation bias, we present in Table 3 four OLS models corresponding to the first prediction (first wave) and each second-chance prediction (second, third and fourth waves). We regress subjects' underestimation length on sociodemographic variables, personality traits, and global life satisfaction.

**Table 3:** OLS models.

	**Under–estimation** **1st wave**	**Under–estimation** **2nd wave**	**Under–estimation** **3rd wave**	**Under–estimation** **4th wave**
Neuroticism	−0.0741 (0.0848)	−0.288 (0.461)	−0.144 (0.0915)	−0.145 (0.115)
Extraversion	−0.0996 (0.0911)	−0.138 (0.442)	−0.109 (0.0937)	0.175 (0.128)
Openness	−0.0180 (0.0905)	0.260 (0.426)	0.0631 (0.0984)	0.145 (0.128)
Agreeableness	0.0933 (0.106)	−0.646 (0.572)	−0.272^**^ (0.116)	−0.0512 (0.177)
Consciousness	−0.0723 (0.0852)	0.410 (0.322)	−0.0223 (0.0919)	0.101 (0.144)
Age	−0.0012 (0.0580)	0.125 (0.2420)	−0.0191 (0.0566)	0.0791 (0.0803)
Gender	−4.399^***^ (1.285)	4.822 (5.482)	−0.471 (1.376)	−1.681 (1.885)
Education	0.380 (0.672)	−1.273 (2.961)	1.160 (0.708)	0.924 (0.908)
SWLS-1	1.086 (0.877)	4.295 (4.233)	−1.307 (1.034)	0.327 (1.266)
SWLS-2	0.146 (0.856)	−0.0748 (4.087)	0.314 (0.939)	0.127 (1.263)
SWLS-3	−1.299 (1.099)	−5.905 (4.309)	0.980 (1.314)	−1.125 (1.897)
SWLS-4	0.727 (0.760)	−1.565 (3.349)	0.0092 (0.861)	0.205 (1.051)
SWLS-5	−0.0700 (0.580)	−1.035 (2.520)	0.570 (0.599)	−0.0949 (0.820)
Constant	45.71^***^ (6.863)	51.10 (37.06)	34.22^***^ (7.492)	0.830 (10.84)
Observations	1134	69	329	142
R-squared	0.020	0.121	0.046	0.110

*Dependent variable: Under-estimation*.

*SWLS-1: In most ways my life is close to my ideal; SWLS-2: The conditions of my life are excellent; SWLS-3: I am satisfied with my life; SWLS-4: So far I have gotten the important things I want in life; SWLS-5: If I could live my life over, I would change almost nothing*.

*Standard errors in parentheses*.

*** *p < 0.01*,

** *p < 0.05*,

* *p < 0.1*.

*OLS model Under-estimation 1st wave F statistic (13, 1120): 1.72. OLS model Under-estimation 2nd wave F statistic (13, 55): 0.58. OLS model Under-estimation 3rd wave F statistic (13, 315): 1.17. OLS model Under-estimation 4th wave F statistic (13, 128): 1.22*.

We obtain that the under-estimation bias is not related to personality traits for first and second predictions (for all waves)^13^. Moreover, global life satisfaction has no effect on lockdown underestimation. Regarding sociodemographic characteristics, only gender has a significant effect on the first prediction. Specifically, initially men underestimate the duration of the lockdown to a significantly larger extent than women.

Now we focus on participants' willingness to be tested for COVID-19. First, we are interested in the determinants of accepting to be tested for free by the health authorities assuming a 14-day mandatory confinement in case of a positive result. Second, we study the variables affecting subjects' willingness to pay €150 for a private COVID-19 test. Table 4 presents estimates of two Tobit models in which the new variables introduced are the following:

**Table 4:** Tobit models.

	**SQ2**	**SQ3**
SQ1	0.104^*^ (0.0600)	0.232^***^ (0.0899)
Neuroticism	−0.0019 (0.00292)	0.0143^***^ (0.00437)
Extraversion	−0.0050 (0.00319)	0.0124^***^ (0.0046)
Openness	0.00288 (0.0031)	−0.00794^*^ (0.0046)
Agreeableness	0.00397 (0.0036)	0.00865 (0.0054)
Conciousness	−0.00285 (0.00289)	0.00261 (0.0043)
DQ1	−0.0731^*^ (0.0425)	0.0480 (0.0636)
DQ2	0.0233 (0.0417)	−0.0395 (0.0624)
Gender	0.0912^**^ (0.0431)	0.0417 (0.0645)
Education	−0.0071 (0.0225)	0.0019 (0.0336)
SWLS-1	0.0255 (0.0299)	0.0059 (0.0448)
SWLS-2	0.0472 (0.0287)	0.0054 (0.0430)
SWLS-3	0.0210 (0.0379)	0.0356 (0.0567)
SWLS-4	−0.0007 (0.0260)	0.0574 (0.0389)
SWLS-5	−0.0057 (0.0198)	0.0370 (0.0296)
Age	0.00318 (0.0019)	0.000583 (0.0029)
Chronic Disease	0.0493 (0.0787)	−0.0134 (0.118)
Health condition	−0.0504 (0.0744)	0.0233 (0.111)
Home Surface	7.46e-05 (0.0001)	0.0004^*^ (0.0001)
Constant	3.369^***^ (0.298)	0.167 (0.446)
Observations	969	969

*Dependent variable: willingness to be tested for free or paying €150*.

SWLS-1: In most ways my life is close to my ideal; SWLS-2: The conditions of my life are excellent; SWLS-3: I am satisfied with my life; SWLS-4: So far I have gotten the important things I want in life; SWLS-5: If I could live my life over, I would change almost nothing; DQ1: How satisfied are you with your life in general? DQ2: In general, how is your health? SQ1: Have you developed any symptoms compatible with COVID-19 that could make you think you have been infected?

*Standard errors in parentheses*.

*** *p < 0.01*,

** *p < 0.05*,

* *p < 0.1*.

*Tobit model SQ2 Wald test (19, 950): 1.30. Tobit model SQ3 Wald test (19, 950): 2.87*.

- DQ1: daily reported life satisfaction. Ranges from 1 to 5.
- DQ2: daily reported health condition (DQ2): Ranges from 1 to 5.
- SQ1: semiweekly reported symptoms compatible with COVID-19. Ranges from 1 to 4.
- SQ2: dependent variable. Willingness to be COVID-19 tested for free. Ranges from 1 to 4.
- SQ3: dependent variable. Willingness to be COVID-19 tested paying €150 (privately). Ranges from 1 to 4.

Not surprisingly, our results show that people who have developed symptoms compatible with COVID are more willing to be tested in both modalities proposed. However, reported health condition and chronic disease do not affect a subject's willingness to be tested. Also, subjects who report a higher life satisfaction level are less willing to be tested under a mandatory confinement than when the confinement decision is voluntary. People living in larger homes are more willing to have the private test, probably because they are also wealthier. Women are more prone than men to be tested, when the test is free, but not so if they have to pay and be tested privately. Some personality traits matter. Neurotic subjects are more willing to pay in order to be tested. A similar effect is found for Extraversion and Openness, although in the latter case the relationship is weaker.

## Discussion

This study reports results on daily life satisfaction reported by a panel of adults over the entire period of lockdown in the first wave of the COVID-19 pandemic in Spain.

Our study presents some strengths and undoubtedly several limitations. Among the major strengths, (1) we collected data on a daily basis during the entire period of the Spanish lockdown and (2) we incentivized subjects in order to elicit predictions about the end of the lockdown. Monetary incentives have their effect through their influence on cognitive effort, motivational focus and emotional triggers. In their meta-analysis Camerer and Hogarth (1999) claim that financial incentives affect performance in judgment tasks where effort responds to incentives. However, truthful expectations reporting is only assured if all forecasters are paid according to their accuracy. Since only a limited (randomly selected) number of successful forecasters are monetarily rewarded, incentives could potentially introduce a sort of gamble where participants could be favored to report extreme beliefs in which the probability of being rewarded would be more plausible (given that the number of subjects choosing this type of option is presumably lower)^14^.

Among the limitations, our sample is not fully representative of the Spanish population in terms of gender, age, education and location. Regarding gender, more females (60%) than males (40%) responded the survey. Furthermore, the average age of participants in this study is under 30 years old, and more than half of the respondents have got a university degree. In terms of location, our respondents were concentrated in the Spanish region “Comunidad Valenciana” (96%), being 79% from the province of Castellón. All of the biases of the sample relate to the characteristics of our broader subject pool. However, this has made it feasible to maintain a relatively large sample of respondents engaged through a rather long and stressing period, obtaining a dynamic picture of daily life satisfaction during the COVID-19 lockdown in Spain.

On average, self-reported levels of daily life satisfaction weakly increased during the lockdown. Our analysis focuses on lockdown-end expectations, as well as personality and individual factors that can partially explain differences in reported daily life satisfaction during the lockdown. To this end, using monetary incentives we elicited subjects' expectations on the lockdown length as well as sociodemographic characteristics and personality traits.

Given the positive effect of longer lockdown expectations on daily life satisfaction, our results suggest that governments should avoid creating false expectations for a shorter lockdown. This concerns the choice between dictating a lockdown “until necessary” and fixing a particular end-day, which might be postponed later. Whereas Hubei, China was an example of the former strategy, most of the western countries, like United States, Italy, France, United Kingdom and Spain preferred the latter option. In the Spanish case, the government needed the approval of the Congress of Deputies, in order to declare the state of alarm in March 14 and expand it in 2-week periods. Following prospect theory (Kahneman and Tversky, 1979; Tversky and Kahneman, 1992), prolonging the lockdown after creating the expectation that it would probably end by a certain day (interpreted as a reference point), might generate frustration if the postponement is perceived as a loss with respect to the initial expectation that works like a reference point. This hypothesis is supported by our findings. However, when failed expectations are revised by participants, their new prediction does not affect their average daily life satisfaction. This result suggests that governments should carefully manage expectations in order to avoid undesired emotional reactions to deviations from the original announcements. These findings are in line with the findings of reference-dependence framing effects related to the government's communication strategy in the COVID-19 crisis, obtained by Hameleers (2020)^15^ and Briscese et al. (2020).

Regarding personality traits, from the perspective of the Big Five factor model adopted here^16^, there are three domains affecting a respondent's reported daily life satisfaction level during confinement: Neuroticism, Extraversion, and Agreeableness. Neuroticism can be defined as the tendency to respond with negative emotions to threat, frustration, or loss (Lahey, 2009) and is one of the strongest predictors of life satisfaction^17^ because it significantly affects the perceived psychological costs associated to the pandemic. This hypothesis was also supported by recent studies with German samples by Kroencke et al. (2020) and Modersitzki et al. (2020), suggesting that Neuroticism had predicted more worrying and affective reactivity during the pandemic. Consistently with these results and controlling for socio-demographical factors, we find that subjects exhibiting higher levels of Neuroticism report a lower average daily life satisfaction level during confinement.

Regarding Extraversion, although it can be hypothesized that less extraverted subjects can experience higher psychological costs associated to the COVID-19 lockdown than their more extraverted counterparts, the existing evidence is mixed. Modersitzki et al.'s (2020) results suggest that more extraverted subjects present a more negative appraisal of the pandemic. Surprisingly, Folk et al. (2020) obtain that the supposed negative effect of Extraversion reverses (for a Canadian university sample) or disappears (for a sample with mostly American and British adults). Wei's (2020) results show that lower extroversion predicts more severe loneliness, anxiety, and depression during confinement. In the present study, subjects scoring higher in Extraversion report higher daily life satisfaction during confinement.

Finally, we focus on Agreeableness. Since this personality trait is usually related to pro-sociality and kindness, it is expected to yield a higher understanding and acceptance of the need for the restrictions imposed during a lockdown^18^. Consistent with this hypothesis, our results are in line with those obtained by Gupta and Parimal (2020) suggesting a significant positive relationship between Agreeableness and psychological well-being during the lockdown.

Policy-makers usually seek the immediate reward from announcing a short horizon for the crises they handle rather than a more realistic and longer horizon. Our findings illustrate the caveats of such an approach. The shorter the expectation the more negative the psychological impact on citizens. A more responsible and efficient management of expectations favors those who do not foresee that the crisis will not end too soon. From a decision-making point of view, this sounds like the usual choice between short and long term benefits. The present study emphasizes the benefits from longer or at least more realistic expectations. Although the final decision depends on the policy-maker's time preferences it is the collective welfare that sets the optimal time horizon. We have seen that our sample, has systematically underestimated the length of the lockdown. Therefore, we do not find evidence of “crowd wisdom” in forecasting future events (Surowiecki, 2005) implying that if any informational advantage is available to the government, this should be used in favor of longer end-of-crisis forecasts.

## Data Availability Statement

The raw data supporting the conclusions of this article will be made available by the authors, without undue reservation.

## Ethics Statement

The studies involving human participants were reviewed and approved by Laboratorio de Economia Experimental, Universitat Jaume I, Castellón-Spain. The patients/participants provided their written informed consent to participate in this study.

## Author Contributions

GS-G has contributed to the design of the survey and the experiment. He built the review of the literature, and was the main coordinator of the data analysis and the writing of the manuscript. AG-G has contributed to the design of the survey and the experiment. She was also involved in the writing of the final version of the manuscript. NG had the original idea of the study and has contributed to the design of the survey and the experiment. He was also involved in the writing of the final version of the manuscript. NH-Z has contributed to the final design, managed and created the questions, collected the data daily and analyzed the data. She built the tables and figures and was involved in the writing of the final version of manuscript. All authors contributed to the article and approved the submitted version.

## Conflict of Interest

The authors declare that the research was conducted in the absence of any commercial or financial relationships that could be construed as a potential conflict of interest.

## Footnotes

^1^On October 25, a new state of alarm was declared for the country, limiting nighttime mobility and socializing, in order to fight the second wave of COVID-19.

^2^See Brooks et al. (2020) for a review of 24 studies documenting the psychological impact of quarantine.

^3^Qiu et al. (2020) and Meng et al. (2020) show how elderly population can be highly psychological affected by COVID-19.

^4^This is the most common method to study the psychology of everyday life [see Kahneman et al. (2004)].

^5^Data available via OSF at https://osf.io/a6u74/.

^6^This procedure allowed us to enhance retention over the confinement (64.41% of the sample answered 50% of the days, 51.73% of the sample answered 75% of the days, 40.57% of the sample answered 90% of the days).

^7^See Weisberg et al. (2011), regarding gender differences in personality traits based on the Big Five.

^8^In phase 0 (preparation) different timetables were established to walking and exercise with area restrictions, and small businesses were allowed to open for clients booking in advance. In phase 1 (initial) the following activities were allowed: social contact within the same city between people who are not considered to be at risk; car travel with people living with you; opening of sidewalk cafes at 30% of their usual capacity. In phase 2 (intermediate) the following activities were allowed: enter inside bars and restaurants, cinemas, theaters, auditoriums and cultural events up to 30% of their capacity. In phase 3 (advanced) mobility restrictions were relaxed. The aforementioned capacity limit was raised up to 50% and the number of people allowed in social gatherings increased. Visits to senior homes were allowed.

^9^Briscese et al. (2020) find that subjects' reported intention to comply with confinement measures diminishes significantly in case of unmet expectations.

^10^Using personality-trait tests, the authors to the Good Judgment Project aim to select forecasting participants with less cognitive bias than the average subject. We do not find significant differences in any personality domain between good and bad forecasters. *P*-values using a Mann-Whitney test corresponding to each domain comparison are 0.2180 (neuroticism), 0.3287 (extraversion), 0.4809 (agreeableness), 0.9785 (consciousness), and 0.9587 (openness).

^11^It must be noted that this new prediction was elicited before the Spanish government revealed the de-escalation plan on the 28 of April 2020.

^12^At this point, new predictions were affected by a previous announcement of the de-escalation four-phase plan. Figure 2C juxtaposes old and new predictions.

^13^All domains present non-significant effects for all prediction waves, with the exception of Agreeableness in the third wave.

^14^See Witkowski et al. (2018) for designing truthful forecaster selection mechanisms.

^15^The authors show that framing the governmental communication about pandemic in terms of gains instead of losses may be more effective in promoting support for interventions to combat the COVID-19 pandemic.

^16^Stadler et al. (2020) find that psychological responses to the COVID-19 outbreak in a U.S. sample depend on their levels of Neuroticism, Openness, Agreeableness, and Conscientiousness.

^17^See the meta-analysis conducted by Steel et al. (2008).

^18^Jørgensen et al. (2020) state that, among all personality traits, Agreeableness is the most important predictor of support to lockdown restrictions.
